# Southern Indiana Dental Society

**Published:** 1897-01

**Authors:** 


### Southern Indiana Dental Society.

The dentists of Evansville have organized a Dental Society. It is intended for the advancement of dentistry, as well as a social feature. Meetings are to be held twice a month.

We think, from the material in close vicinity of Evansville, to make a tri-State Society including northern Kentucky, southern Illinois and southern Indiana should be one of the best societies in the Middle States. The officers elected are President, F. Hutchinson; Vice-President, F. A. Raymond; Secretary, M. M. Haas; Treasurer, S. F. Jacobi.

Papers, at the next meeting, will be read by Drs. C. E. Pittman, M. M. Haas, S. F. Jacobi and Max H. Martin.

